# Effect of continuous positive airway pressure therapy on a large hemangioma complicated with obstructive sleep apnea syndrome: a case report

**DOI:** 10.1186/1752-1947-4-271

**Published:** 2010-08-12

**Authors:** Maria Antoniadou, Paschalis Steiropoulos, Evangelia Serasli, Venetia Tsara

**Affiliations:** 1 Sleep Unit, 2nd Chest Department, General Hospital "G. Papanikolaou", Exohi 57010, Thessaloniki, Greece

## Abstract

**Introduction:**

Hemangiomas involving the upper airway can be an uncommon cause of obstructive sleep apnea syndrome.

**Case presentation:**

A 26-year-old Caucasian man with a known history of a large hemangioma of his head and neck presented with sleep-disordered breathing to the sleep unit of our hospital. Severe obstructive sleep apnea syndrome was revealed on polysomnography. Nasal continuous positive airway pressure was implemented effectively, reducing daytime hypersomnolence and significantly improving sleep parameters. After three years of adherent use, the patient remains in a good condition and the hemangioma is stable.

**Conclusion:**

Application of continuous positive airway pressure can be an effective treatment for patients with obstructive sleep apnea syndrome complicated with vascular tumors. Periodic follow-up of these patients is necessary, as little is known about the long-term effects of continuous positive airway pressure therapy.

## Introduction

Obstructive sleep apnea syndrome (OSAS) is the most common sleep-related breathing disorder, with a worldwide prevalence of 4 and 2% in middle-aged men and women, respectively [1]. The clinical manifestations in patients with this condition include daytime hypersomnolence, neurocognitive dysfunction, cardiovascular disease and metabolic disorders [2]. There are many risk factors for the development of OSAS including alterations in upper airway anatomy in some patients, which predisposes them to upper airway obstruction by increasing pharyngeal collapsibility [3]. An uncommon cause of anatomic narrowing of the upper airway is soft tissue tumors of the head and neck.

We report the case of a patient with a large head and neck hemangioma complicated by the presence of OSAS, which was successfully treated using continuous positive airway pressure (CPAP).

## Case presentation

**Figure 1:**
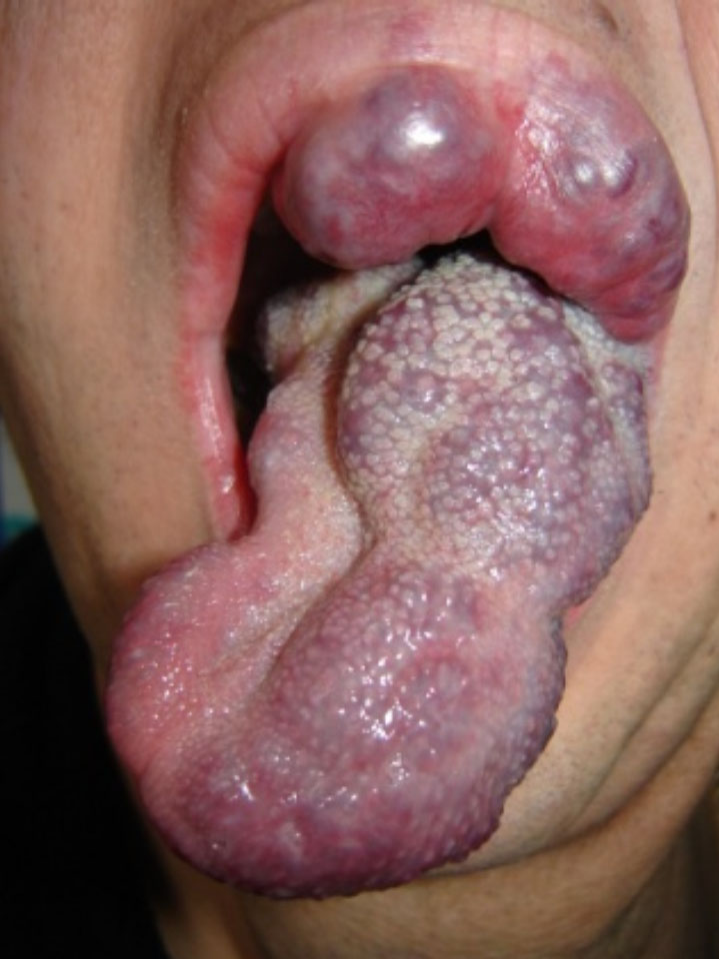
**Involvement of the tongue and oral cavity in our patient with a large head and neck hemangioma**.

A 26-year-old Caucasian man presented to the sleep unit of the General Hospital "G. Papanikolaou", Thessaloniki, Greece, with daytime somnolence, fatigue, and loud snoring. He had been involved in a near-fatal motor vehicle accident four years previously caused by sleepiness while driving. His mother reported the occurrence of apneic events during the night. He had a past medical history of a congenital hemangioma involving his left temporal region, and the left half of his face, oral cavity, tongue and pharynx. The hemangioma extended into the left side of his neck (Figure 1). In the preceding 10 years, the patient had undergone transarterial catheter embolization five times with no significant improvement.

**Figure 2:**
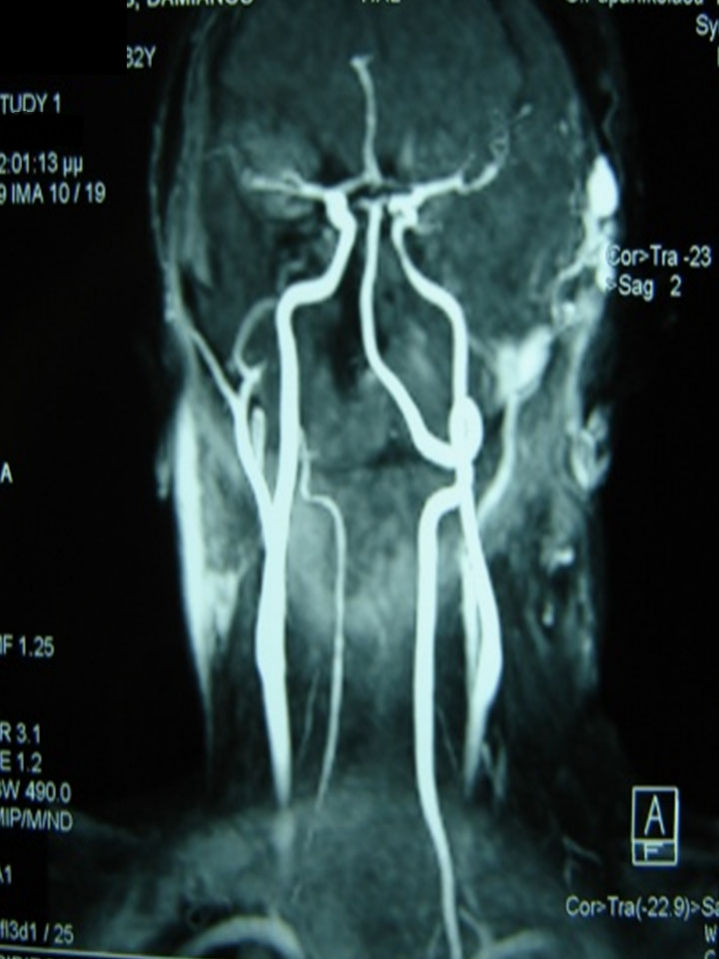
**Vascular mass with two feeding arteries (external carotid and middle cerebral artery) as shown on a magnetic resonance angiogram**.

A physical examination revealed that his body mass index was about 23.3 kg/m^2 ^and his neck circumference was 44 cm. His Epworth Sleepiness Scale (ESS) score was 17 and a otolaryngologic evaluation reported that the hemangioma involved the left half of his tongue, uvula, and soft palate and his left nasal concha, with a high Mallampati score (Class 4). In order to evaluate the extent of his lesion, magnetic resonance angiography was performed and a vascular mass that was fed from both the external carotid and middle cerebral arteries was found (Figure 2). The polysomnographic sleep study revealed that the patient had severe OSAS with an Apnea-Hypopnea Index (AHI) of 60 events/hour and a minimum oxygen saturation of 58% (Table 1).

**Table 1:** Polysomnographic parameters and ESS before and after one year on continuous positive airway pressure therapy

	Before CPAP	After CPAP
AHI (events/hour)	60	1
Mean SpO_2 _(%)	84	96.3
Min SpO_2 _(%)	58	87
Time SpO_2 _< 90% (%TST)	96.7	0.1
ESS	17	3

Abbreviations: AHI = apnea hypopnea index; CPAP = continuous positive airway pressure; ESS = Epworth Sleepiness Scale; SpO_2 _= pulse oxymetric saturation; TST = total sleep time.

The following night, CPAP via a nasal mask was applied to the patient and titration of optimal pressure was determined at a second sleep study. At 10 cm of water pressure of nasal CPAP, the patient's snoring was eliminated, his AHI decreased considerably, and both his oxygen saturation and sleep efficiency improved (Table 1). The application of CPAP was well tolerated by the patient and no complications were observed. Therefore, CPAP treatment was prescribed and the patient was followed up once a year for three years. At the one-year follow up, the patient noted significant improvement in his cognitive performance and daytime functions. His ESS score had declined to three. No symptoms, such as headache or swelling of the hemangioma, were reported. After three years, the patient compliantly uses the nasal CPAP treatment (mean use of more than four hours/night) and both his daytime alertness and sleep effectiveness are significantly improved. The status of his hemangioma remained unchanged.

## Discussion

OSAS is a highly prevalent disease accompanied by major comorbidities. Structural changes, such as tonsilar hypertrophy, retrognathia, macroglossia, adenoid tissue and variations in craniofacial features, promote the occurrence of apneas and hypopneas due to anatomic narrowing of the upper airway. In selected patients, surgical intervention may have beneficial effects [2]. Uncommon causes of structural alterations in the pharyngeal wall, oral cavity and tongue are benign tumors and cysts, which are responsible for 1.5% of cases of OSAS [4].

Hemangioma is a benign vascular tumor that occurs in 1.1 to 2.6% of newborn babies [5]. In 60 to 70% of cases, hemangiomas are localized to the head and neck. However, all other parts of the body can be affected. This lesion can be complicated with ulceration, infection, bleeding, ocular involvement, disfigurement and heart failure [6]. If the central respiratory tract is involved, hemangioma may lead to life-threatening obstruction. The diagnosis of a hemangioma is usually made on the basis of a patient's history and clinical findings. Ultrasonography is a useful imaging technique for the evaluation of hemangiomas. However, magnetic resonance imaging is the ideal tool, providing a more specific diagnosis. When accompanied by a magnetic resonance angiography, magnetic resonance imaging provides information not available from other non-invasive techniques, accurately determining the extent of the hemangioma [7,8]. When complications are present, a medical or surgical intervention is usually recommended [9-11].

In the current literature, few cases of patients with hemangiomas associated with OSAS have been reported. Kimura *et al*. [12] presented three cases of patients with OSAS due to mucosal hemangiomas of the oral cavity, which were successfully treated with the use of CPAP. All three patients were compliant with nightly use of nasal CPAP and at follow up their hemangiomas remained stable. Becker *et al*. [13] reported the case of a patient with OSAS and congenital temporal and cervical hemangiomas, who developed severe headache and internal hydrocephalus while using nasal bilevel positive airway pressure ventilation. After other possible diagnosis had been excluded, the cause of the patient's headaches was postulated to be the artificial respiration-dependent swelling of the subcutaneous temporal and cervical hemangiomas. A rare case of an 18-month-old child with sleep apnea and recurrent epistaxis due to an ethmoidal hemangioendothelioma was described by Semino *et al*. [14]. Hemangioendotheliomas are tumors that have a histology resembling somewhere between a hemangioma and an angiosarcoma. In that patient, a presurgery embolization was performed and was followed by endoscopic resection of the tumor. After four years, no sign of disease recurrence was observed. Recently, Thong *et al*. [15] reported the case of a patient with symptoms suggestive of OSAS due to an uvular hemangioma. The symptoms resolved after a complete excision of the lesion using a carbon dioxide laser.

In this case, for the patient having a large non-operable hemangioma and severe OSAS, responsible for daytime sleepiness and an increased risk of several types of morbidity, the use of CPAP seemed a logical approach.

## Conclusions

Hemangiomas may be complicated with severe OSAS when the upper airway is involved. Close monitoring and prompt diagnosis of the patient with OSAS are required, especially for patients with enlarging hemangiomas. CPAP use is an effective and well-tolerated solution. However, periodic follow up is required, because the long-term outcomes of the implementation of CPAP on vascular lesions are unknown.

## Consent

Written informed consent was obtained from the patient for publication of this case report and accompanying images. A copy of the written consent is available for review by the Editor-in-Chief of this journal.

## Competing interests

The authors declare that they have no competing interests.

## Authors' contributions

MA and PS contributed to the diagnosis of the patient and to the writing of the paper. ES contributed to the diagnosis and treatment of the patient. VT contributed to the diagnosis and treatment of the patient and has been responsible for his follow-up examinations. All authors read and approved the final manuscript.

## References

[B1] Young T Palta M Dempsey J Skatrud J Weber S Badr S The occurrence of sleep-disordered breathing among middle-aged adults N Engl J Med 1993 328 1230 1235 10.1056/NEJM199304293281704 8464434

[B2] White DP Sleep apnea Proc Am Thorac Soc 2006 3 124 128 10.1513/pats.200510-116JH 16493160

[B3] Patil SP Schneider H Schwartz AR Smith PL Adult obstructive sleep apnea: pathophysiology and diagnosis Chest 2007 132 325 337 10.1378/chest.07-0040 17625094 PMC2813513

[B4] Sher AE Obstructive sleep apnea syndrome: a complex disorder of the upper airway Otolaryngol Clin North Am 1990 23 593 608 2199896

[B5] Drolet BA Esterly NB Frieden IJ Hemangiomas in children N Engl J Med 1999 341 173 181 10.1056/NEJM199907153410307 10403856

[B6] Smolinski KN Yan AC Hemangiomas of infancy: clinical and biological characteristics Clin Pediatr 2005 44 747 766 10.1177/000992280504400902 16327961

[B7] Kesava PP Turski PA MR angiography of vascular malformations Neuroimaging Clin N Am 1998 8 349 370 9562593

[B8] Vilanova JC Barceló J Villalón M MR and MR angiography characterization of soft tissue vascular malformations Curr Probl Diagn Radiol 2004 33 161 170 10.1016/j.cpradiol.2004.04.001 15306760

[B9] Willenberg T Baumgartner I Vascular birthmarks Vasa 2008 37 5 17 10.1024/0301-1526.37.1.5 18512538

[B10] Poetke M Philipp C Berlien HP Flashlamp-pumped pulsed dye laser for hemangiomas in infancy: treatment of superficial vs mixed hemangiomas Arch Dermatol 2000 136 628 632 10.1001/archderm.136.5.628 10815856

[B11] Cholewa D Waldschmidt J Laser treatment of hemangiomas of the larynx and trachea Lasers Surg Med 1998 23 221 322 10.1002/(SICI)1096-9101(1998)23:4<221::AID-LSM5>3.0.CO;2-A 9829433

[B12] Kimura K Adlakha A Staats BA Shepard JW Jr Successful treatment of obstructive sleep apnea with use of nasal continuous positive airway pressure in three patients with mucosal hemangiomas of the oral cavity Mayo Clin Proc 1999 74 155 158 10.4065/74.2.155 10069354

[B13] Becker R Schäfer H Bauer BL Intracranial pressure in sleep apnea, hydrocephalus and congenital hemangioma. A case report Zentralbl Neurochir 1994 55 63 68 8053280

[B14] Semino L Pagella F Delù G Todeschini A Luinetti O Zappoli F Castelnuovo P Endoscopic treatment of ethmoidal hemangioendothelioma: case report and review of the literature Am J Otolaryngol 2006 27 287 290 10.1016/j.amjoto.2005.11.006 16798411

[B15] Thong JF Pang KP Siow JK Hemangioma of the uvula causing loud habitual snoring - a rare entity Med J Malaysia 2008 63 408 409 19803302

